# A Document-Based Comparative Analysis of Physical Infrastructure Planning in Pathology Laboratories

**DOI:** 10.5146/tjpath.2026.15090

**Published:** 2026-09-15

**Authors:** Mustafa Huz

**Affiliations:** Department of Medical Pathology, Inonu University, Faculty of Medicine, Malatya, Türkiye

**Keywords:** Pathology laboratory, Laboratory planning, Laboratory infrastructure, Ventilation systems

## Abstract

Objective: Medical pathology laboratories have a long-standing tradition while continuously evolving through the integration of traditional and modern diagnostic techniques. Today, rapidly evolving and transforming laboratories require renewal in organizational, infrastructure, and managerial approaches. The aim of this study is to conduct a document-based comparative analysis of physical infrastructure criteria related to pathology laboratories and to examine how convergent and divergent standards are reflected in critical planning decisions.

Material and Methods: A document-based comparative analytical design was applied using predefined infrastructure parameters. International guidelines, technical standards, and selected scientific publications, published between January 1, 2000, and April 30, 2025, addressing pathology laboratories or laboratory environments involving chemical and biological risks were included. Infrastructure-related criteria were systematically extracted, classified, and compared across predefined domains to identify areas of convergence, divergence, and indeterminate guidance.

Results: Core safety principles-including controlled airflow direction, negative pressure relationships, source-based vapor capture, and contamination-resistant surface materials-demonstrated strong cross-document alignment. In contrast, variability emerged in numeric thresholds and implementation models, particularly for air change rates, lighting parameters, and selected environmental comfort indicators. These variations directly affected planning flexibility, renovation sequencing, and risk-based infrastructure decisions.

Conclusion: Physical infrastructure planning in pathology laboratories requires contextual interpretation of international standards rather than direct transfer of prescriptive thresholds. A flexible, risk-informed planning approach is essential for sustainable and operationally resilient laboratory environments.

## INTRODUCTION

Medical laboratories, and pathology laboratories (PL) in particular, are technically complex environments in which diagnostic accuracy, occupational safety, and operational continuity are structurally interdependent. The design, construction, and renovation of such facilities therefore involve multidimensional decisions affecting spatial organization, mechanical systems, regulatory compliance, and long-term institutional capacity. Laboratory infrastructure cannot be evaluated solely on technical adequacy; it must sustain safe operation, accommodate evolving diagnostic technologies, and remain adaptable within institutional and regulatory constraints (1–4).

Renovation of pathology laboratory spaces most commonly results from operational pressures, including spatial insufficiency, equipment limitations, increasing diagnostic workload, workforce expansion, and technological integration. Regulatory updates, occupational safety requirements, and environmental standards frequently necessitate modification of ventilation systems, electrical installations, and fire safety provisions. Progressive material degradation and incremental system modifications further contribute to infrastructure obsolescence (4–7). These drivers underscore that laboratory renewal is rarely elective; it is typically reactive to accumulated structural and regulatory demands.

However, renovation processes are inherently constrained. Laboratory location within the healthcare facility, compliance with environmental and occupational regulations, fire protection strategies, existing mechanical infrastructure, and prior architectural decisions substantially limit design flexibility (2,6–9). As a result, standardized solutions cannot be directly transferred across institutions without contextual adaptation.

Despite these constraints, both new construction and infrastructure renewal can be systematically planned to meet architectural, technical, and safety requirements while preserving functional integrity and long-term adaptability. Planning decisions must consider anticipated diagnostic scope, technological evolution, and institutional strategy rather than immediate operational capacity alone. At the same time, accumulated professional standards and safety regulations define non-negotiable baseline principles that apply across pathology laboratories regardless of scale or subspecialization (2,3,10).

Effective infrastructure planning therefore requires active involvement of pathology leadership. Although infrastructure design extends beyond routine diagnostic responsibilities, its long-term consequences directly affect diagnostic reliability, occupational risk, and operational resilience. Infrastructure planning should be understood as a strategic process shaping future laboratory capacity rather than a short-term technical intervention.

Within this framework, a critical gap persists: international guidelines, standards, and technical documents addressing laboratory infrastructure remain fragmented and heterogeneous, and their implications for practical planning decisions are rarely examined through a systematic analytical lens. The present study addresses this gap by applying a document-based comparative analytical framework to predefined infrastructure parameters, evaluating areas of convergence, divergence, and indeterminate guidance to inform evidence-based pathology laboratory establishment and renewal.

## MATERIALS and METHODS

### Study Design

This study applied a document-based comparative analytical design to evaluate physical infrastructure requirements for pathology laboratories in both new-build and renovation contexts. A two-phase methodology was implemented: an exploratory mapping phase informing parameter development, followed by a formal analytical phase generating the comparative dataset.

The primary research question was: What minimum consensus-driven physical infrastructure parameters can be identified across international normative and technical documents to inform evidence-based planning and renovation of pathology laboratories?

### Search Strategy

Rather than a conventional bibliographic systematic review, a structured screening approach tailored to normative and technical infrastructure documents was applied.

A predefined keyword framework guided document identification in both phases. Core search terms included:

• Pathology laboratory infrastructure

• Laboratory design and construction

• Biosafety level (BSL) laboratories

• HVAC systems in medical laboratories

• Laboratory ventilation standards

• Pressure differential in healthcare facilities

• Negative pressure laboratory design

• Laboratory zoning design (clean/dirty areas)

• Specimen workflow laboratory design

• Contamination control in laboratories

• Histopathology laboratory planning

• Molecular laboratory layout

• Occupational noise exposure in laboratories

• Lighting standards for healthcare facilities

• Digital pathology infrastructure

• Whole-slide imaging systems

Searches were conducted across institutional repositories, standards organizations, governmental health authorities, professional societies, and technical guidance platforms, prioritizing operational criteria and measurable thresholds.

### Data Set Creation and Document Selection

Documents published between January 1, 2000, and April 30, 2025 addressing physical infrastructure requirements in pathology or chemical/biological risk laboratories were screened. Documents outside this period were excluded from formal analysis but could be referenced contextually.

Eighty text-based documents were identified. After removal of four duplicates using hash verification, 76 unique documents underwent full-text assessment. Thirty-eight were excluded due to insufficient operational specificity or lack of direct relevance. The final analytical dataset comprised 38 documents (Table 1).

**Table 1:** Structured Document Screening and Selection Process

**Stage**	**Number of Documents**
Initial identified records	80
Duplicates removed	4
Records screened (full-text)	76
Excluded after eligibility review	38
Final analytical dataset	38

The exploratory phase additionally reviewed 52 independent web domains to support parameter development. Detailed exclusion logs and exploratory domain lists are provided in Supplementary Tables SI and SII.

### Data Sources

The final dataset included international technical standards, health facility planning guidelines, occupational safety regulations, laboratory biosafety manuals, accreditation frameworks, engineering reference works, and peer-reviewed publications addressing infrastructure-related operational criteria. The selected documents represent widely cited international reference frameworks that are frequently used in laboratory planning and infrastructure guidance.

### Eligibility Criteria

Inclusion Criteria

Documents meeting at least one of the following:

• Explicit applicability to pathology laboratories or laboratory environments with chemical/biological risks

• Provision of a numerical threshold, technical definition, or planning principle related to physical infrastructure

• Direct relevance to ventilation, pressure regimes, lighting, noise, spatial dimensions, surface materials, safety equipment, or IT/digital pathology infrastructure

• Publication by a recognized institution/organization or academic publisher

Exclusion Criteria

Documents meeting at least one of the following were excluded:

• Local administrative regulations lacking infrastructure-level technical criteria

• Texts limited to aspirational statements without operational thresholds/definitions

• Guidance limited to office/administrative areas without pathology-specific risk considerations

• Opinion-based publications, non-peer-reviewed commentaries, and informal web/blog content lacking operational infrastructure criteria

### Analytical Parameters

Infrastructure criteria were classified under nine predefined parameters:

1. Infrastructure resilience and continuity

2. Location and layout typology

3. Air change rates

4. Pressure regime and airflow direction

5. Chemical vapor control and local exhaust solutions

6. Floor/wall/surface materials

7. Emergency eye-wash stations and safety equipment

8. IT and digital pathology infrastructure

9. Human-infrastructure interaction

### Comparative Analysis

Each included document was systematically reviewed against the nine predefined analytical parameters prior to categorical classification. For each parameter, documents were coded as fully aligned, partially aligned, divergent, or indeterminate based on predefined analytical criteria evaluating threshold consistency and implementation logic.

The dataset comprised heterogeneous normative document types. Only documents providing measurable criteria or enforceable planning principles were included in formal comparison, enabling cross-category synthesis independent of document typology (Table 2).

**Table 2:** Normative Categories Represented in the Analytical Dataset (n=38)

**Normative Category**	**Examples in Dataset**	**Analytical Role**
Technical ventilation & HVAC standards	ASHRAE 170; UNC; Siemens	Numerical thresholds (ACH, pressure, airflow)
Health facility planning guidelines	NHS HBN 15; iHFG; FGI	Spatial organization and zoning principles
Laboratory design reference works	NRC; DiBerardinis	Integrated infrastructure planning logic
Biosafety & occupational safety documents	WHO LBM; OSHA	Risk containment and exposure limits
Accreditation & quality frameworks	ISO 15189; CAP	Performance-based infrastructure implications
Digital pathology standards	DICOM Suppl. 145; FDA	IT system architecture & interoperability

Coding was performed using predefined parameter definitions to ensure methodological consistency. Where classification required interpretative judgment, the rationale is described in the Results and Discussion sections rather than within tables.

### Ethical Considerations

This study received approval from the İnönü University Scientific Research and Publication Ethics Committee (Decision No: 2026/9206; January 13, 2026). The study involved analysis of publicly available normative and technical documents and did not include human or animal subjects.

## FINDINGS

### Infrastructure Resilience and Continuity

In this study, infrastructure resilience and continuity are examined not in relation to the structural safety of pathology laboratories (PL), but with respect to the uninterrupted, controllable, and secure operation of critical technical infrastructure components, including HVAC systems, energy supply, information technologies, and security systems. Comparative evaluation of international guidelines indicates that resilience is consistently framed not as a single technical intervention, but as the integrated management of design, operation, maintenance, and emergency scenarios, emphasizing system performance under both routine and non-routine conditions (2,4–6,10,11).

Within healthcare laboratory settings, continuity is analytically linked to the predictability and controllability of system behavior and the capacity to sustain services during abnormal operating conditions, including emergency scenarios. The comparative analysis further demonstrates that the AusHFG and iHFG documents extend the concept of continuity beyond technical infrastructure protection to encompass the maintenance of service capacity under conditions of workload fluctuation, infection risk, and extraordinary operational demands (3,7,10–13)

Accordingly, PL infrastructure resilience and continuity emerge not as static, building-level safety attributes, but as dynamic performance criteria that directly inform planning, design, and operational decision-making processes across the laboratory lifecycle (Table 3).

**Table 3:** Infrastructure Resilience and Operational Continuity in Pathology Laboratories: A Comparative Analysis

**Infrastructure Parameter**	**Alignment Category**	**Operational Basis**	**Common Approach in Guidelines / Standards**	**Impact on Planning Decisions**	**Representative References**
Prevention of exhaust-intake interaction (re-entrainment risk)	Partially aligned	Principle-based	Exhaust discharge and outdoor air intake configurations are designed to minimize re-entrainment risk; however, explicit distance or velocity thresholds are not consistently specified across documents.	Exhaust shaft/roof placement; outdoor air intake location; building massing; wind effects.	NRC, 2000; NHS, 2005; ASHRAE, 2020
Continuity of exhaust systems	Partially aligned	Principle-based	Continuity of exhaust systems as a critical control measure for hazardous vapors and odors.	Redundancy of exhaust fans; emergency power supply; maintenance access planning.	NRC, 2000; NHS, 2005
Pressure regime continuity	Partially aligned	Mixed (principle + operational cascade logic)	Maintenance of directional airflow and negative pressure in risk-bearing zones under normal and abnormal conditions.	Zoning strategy; pressure cascade design; separation of clean-dirty areas.	ASHRAE, 2020; NHS, 2005
System redundancy and diversity	Partially aligned	Principle-based	Use of diversified and redundant systems to ensure operational continuity during failures or maintenance.	Backup systems; system segregation; failure scenario planning.	NHS, 2005; ASHRAE, 2020
Serviceability and isolation through system placement	Unspecified / indeterminate	Principle-based	Maintainability and system isolation are recommended to enable servicing without operational disruption; however, measurable operational thresholds are rarely explicitly defined.	Maintenance zoning; access routes; service shaft organization.	Griffin, 2007; DiBerardinis et al., 2013

**Note**: Alignment categories were assigned according to predefined analytical criteria described in the Methods section and reflect operational specificity rather than narrative emphasis.

### Planning the Pathology Laboratory and Layout Type

This comparative analysis positions the location and layout type of the pathology laboratory (PL) within the healthcare facility as a determinant planning variable, rather than a purely spatial or architectural preference. The findings demonstrate that decisions related to PL placement and layout configuration directly influence risk management strategies, workflow organization, and infrastructure design-particularly the functional separation of ventilation, pressure, and service systems-as well as long-term adaptability (Table 4) (2,10,12).

**Table 4:** Planning and Layout Typology of Pathology Laboratories: A Comparative Analytical Summary

**Examined Dimension**	**Alignment Category**	**Operational Basis**	**Common Approach in Guidelines / Standards**	**Diverging Approaches**	**Impact on Planning Decisions**	**Source Mapping**
Location within the healthcare facility	Partially aligned	Principle-based	Risk-bearing unit requiring controlled siting and separation.	Access priority vs environmental isolation emphasis.	Separation from main circulation; controlled adjacency.	NRC (2000); NHS (2005); iHFG (2022)
Plan typology classification	Strongly aligned	Principle-based	Open-plan, closed/segmented layouts, and special-purpose units constitute the core classification framework.	Level of typological detail varies across guidelines.	Functional separation prioritized over a single uniform plan type.	NRC (2000); NHS (2005)
Suitability of open-plan layouts	Partially aligned	Mixed (risk-based operational differentiation)	Considered efficient for highly automated units with low chemical risk.	Deemed insufficient for high-risk pathology sub-units.	Separation of macroscopic examination and chemical processing areas from open-plan zones.	NRC (2000); DiBerardinis et al. (2013)
Closed / segmented plan approach	Partially aligned	Mixed (spatial-infrastructure integration logic)	Closed-plan layouts are defined as infrastructure-based safety organizations rather than mere spatial partitions.	Scope and degree of segmentation differ among standards.	Integration of ventilation, pressure, and airflow regimes with spatial planning.	NHS (2005); CLSI (2007)
Risk variability among sub-units	Partially aligned	Mixed (risk-based spatial differentiation logic)	Coexistence of units with differing risk profiles is acknowledged.	Definitions of risk zones vary in detail.	Infrastructure-based separation of areas requiring chemical exposure control.	NHS (2005); iHFG (2022)
Flexible and adaptive planning	Partially aligned	Mixed (future adaptability + continuity logic)	Flexibility without structural modification and modular reconfigurability are recommended.	Scale and implementation level vary by laboratory type.	Maintaining uninterrupted operations during renovation; adaptation to capacity and technology changes.	NHS (2005); NRC (2000); DiBerardinis et al. (2013)
Sustainability and serviceability	Partially aligned	Mixed (service accessibility + long-term continuity logic)	Modular service distribution and infrastructure accessibility are accepted principles.	Implementation details vary across countries and standards.	Long-term renewability and operational continuity.	NHS (2005); DiBerardinis et al. (2013); NRC (2000)

**Note:** Alignment categories were assigned according to the predefined analytical framework described in the Methods section. Classification reflects the level of operational specificity and cross-document consistency rather than the presence of identical design prescriptions.

Comparative evaluation indicates that categorizing medical laboratories into open-plan, closed or partitioned layouts, and dedicated units such as molecular pathology provides a functional framework for PL planning. While open-plan configurations may support efficiency in highly automated settings with relatively limited contamination risk, most PLs necessitate controlled containment and functional separation due to the presence of risk-intensive subunits, including macroscopy and chemical processing areas. Within this context, closed-plan layouts should be interpreted not simply as physical partitions, but as safety-oriented spatial organizations supported by ventilation and exhaust systems, pressure regimes, and dirty-clean workflow separation (7,10,14,15).

Within this planning framework, flexible, adaptable, and sustainable principles constitute the core criteria of an effective laboratory layout. The ability to meet infrastructure requirements without structural modification, the use of modular workbench and equipment configurations, and the accessibility of infrastructure systems emerge as key design attributes. These features enable PLs to maintain operational functionality during renovation processes while supporting future capacity expansion and technological transitions (2,7,10,12).

### Air Change Rates (ACH)

The air change rate (ACH) in pathology laboratories (PL) is not merely a technical determinant of indoor air quality, but a core infrastructure parameter that enables the integrated management of chemical exposure control, pressure regime continuity, and occupational safety. Comparative evaluation of international guidelines demonstrates that ACH cannot be applied as a single, fixed threshold across all PL environments; instead, it must be differentiated according to the functional role and risk profile of individual laboratory subunits (2,10,11).

Across the analyzed documents, a minimum reference value of 6 ACH is widely recognized for general technical laboratory areas. However, this value is consistently framed as a baseline threshold rather than a definitive safety guarantee. Guidelines further emphasize that ventilation effectiveness depends not only on total air exchange but also on the proportion of fresh (outside) air, which is essential for dilution and removal of chemical vapors; reliance solely on recirculated air is considered insufficient for risk control (11,15).

In contrast, microscopy and reporting areas-where chemical exposure risk is absent-are systematically differentiated from technical laboratory zones and evaluated within the scope of office-type environments. For these low-risk areas, ventilation requirements are defined primarily through fresh air flow rates and general indoor air quality criteria rather than laboratory-specific ACH prescriptions, corresponding in practice to approximately 2-4 ACH (7,10,11). This distinction reinforces the principle that ventilation design in PLs must be risk-based and unit-specific.

For subunits with intensive formalin use, particularly macroscopy areas, the reviewed guidelines deliberately avoid specifying a single prescriptive ACH value. Instead, a flexible exposure-oriented approach is adopted. Technical assessments indicate that macroscopy units may present a chemical vapor risk profile comparable to autopsy rooms; accordingly, air change rates in these areas may be increased to up to 12 ACH when warranted by exposure conditions (16,17). These findings confirm that ACH functions as a differentiated planning parameter, rather than a homogeneous laboratory-wide value.

Importantly, the guidelines also caution against an exclusive focus on increasing ACH. High air change rates, when combined with improperly positioned supply and exhaust diffusers, may generate turbulence that facilitates contaminant transport into the breathing zone. Consequently, ACH is consistently addressed within a holistic ventilation strategy that integrates airflow direction, pressure zoning, and local exhaust performance (7,18).

From the perspective of energy efficiency and operational continuity, the comparative analysis further indicates that temporal and functional adaptability of ventilation regimes constitutes a critical planning decision. In areas without chemical exposure risk and during periods of laboratory inactivity, ACH values may be safely reduced-provided that safety conditions are maintained-particularly through the use of variable air volume (VAV) systems (17,18). This approach positions ventilation exchange rates not only as a safety determinant but also as a strategic variable influencing sustainability and operational cost control (Table 5).

**Table 5:** Air Change Rates (ACH) in Pathology Laboratories: A Comparative Analysis

**Evaluated Dimension**	**Alignment Category**	**Operational Basis**	**Common Approach in Guidelines / Standards**	**Diverging Approaches**	**Impact on Planning Decisions**	**Example of Numerical Threshold / Technical Criterion**	**Source Mapping**
General laboratory technical areas	Partially aligned	Prescriptive (numeric threshold)	Minimum ACH required in chemical/biological risk areas	Fixed ACH values versus risk- and function-based approaches	Unit-based zoning; HVAC capacity calculations	≥6 ACH, with at least one-third supplied as outdoor (fresh) air	ASHRAE; Siemens
Grossing rooms and intensive chemical-use areas	Partially aligned	Mixed (prescriptive ACH + performance-based local exhaust logic)	Increased ventilation due to contaminant load	Prescriptive ACH values versus prioritization of local exhaust with ACH treated as secondary	Integrated design of local exhaust and general ventilation systems	Values up to 10-12 ACH (high-risk operational assumption)	AIA; Siemens
Microscopy rooms and reporting areas	Partially aligned	Prescriptive (risk-based numeric threshold)	Office-level ventilation sufficient in low-risk zones	Uniform lab ACH vs task-based differentiation	Energy load, noise levels, and occupant comfort as planning inputs	Typical office values of 2-4 ACH	NHS; CLSI
ACH not as a standalone safety indicator	Fully aligned	Principle-based (multi-parameter ventilation safety logic)	ACH alone insufficient for safety	Persistence of the assumption that “higher ACH equals safety ”	ACH assessed together with airflow direction, diffuser placement, and pressure regime	ACH combined with directional airflow and negative pressure	DiBerardinis et al ; UNC
Energy efficiency and operational continuity	Partially aligned	Mixed (prescriptive minimums + adaptive VAV strategies)	Continuous high ACH is associated with increased energy and operational costs	Fixed ACH operation versus variable air volume (VAV) strategies	VAV systems; night and idle operational scenarios	VAV regimes allowing reduction to as low as 4 ACH in technical areas	AIA; Siemens; ASHRAE
Idle or low-use scenarios	Partially aligned	Mixed (numeric minimum + pressure continuity requirement)	Reduced ACH acceptable under pressure control	Minimum thresholds vary across guidelines	Operational scenarios integrated with HVAC design	Lower limit of 4 ACH while maintaining negative pressure	ASHRAE; NHS

**ACH: **Air Changes per Hour, **HVAC:** Heating, Ventilation, and Air Conditioning, **VAV:** Variable Air Volume, **LEV:** Local Exhaust Ventilation **Note:** Alignment categories were assigned according to predefined analytical criteria described in the Methods section. Classification reflects cross-document consistency in operational specificity and numerical threshold definition.

### Pressure Regime and Air Flow Direction

The PL pressure regime and airflow direction are considered a fundamental infrastructure component in preventing the spread of chemical and biological contaminants to areas outside the laboratory. The reviewed guidelines and standards define maintaining technical areas under negative pressure relative to adjacent areas and directing airflow from clean areas to contaminated areas as a common safety principle. This approach not only ensures contamination control but also allows for the establishment of functional zoning between subunits of the laboratory with different risk levels (4,10,12,14,15). A comparative assessment of pressure regimes reveals differences in the level of definition among the guidelines (Table 6). While some of the reviewed guidelines and standards propose numerical pressure differential ranges, others address the pressure regime more in terms of inter-area relationships, continuity, and traceability. These findings suggest that evaluating the pressure regime solely as a fixed threshold value would be insufficient. It reveals that the pressure regime is a dynamic design input that must be evaluated in conjunction with elements such as airflow direction, spatial zoning, door traffic, and system airtightness during the planning process (4,10,19,20).

**Table 6:** Pressure Regimes and Airflow Direction in Pathology Laboratories: A Comparative Analysis

**Evaluated Dimension**	**Alignment Category**	**Operational Basis**	**Common Approach in Guidelines / Standards**	**Diverging Approaches**	**Impact on Planning Decisions**	**Example of Numerical Threshold / Technical Criterion**	**Source Mapping**
Negative pressure principle	Fully aligned	Principle-based (directional containment logic)	Areas involving chemical/biological risks are maintained under negative pressure relative to adjacent spaces	Degree of negative pressure and zoning scope vary across guidelines	Laboratory-corridor-office relationships; door placement; HVAC zoning	“Laboratory areas should be negative relative to adjacent spaces” (qualitative requirement”).	ASHRAE; NHS; CLSI
Airflow direction (clean-to-dirty)	Fully aligned	Principle-based (risk-hierarchy airflow logic)	Airflow is directed from lower-risk to higher-risk areas as a fundamental principle	Defined as a planning principle versus an operational rule	Spatial hierarchy; entry-exit configuration; number of transitions	Directional airflow principle (no numerical threshold).	NHS; iHFG
Pressure zoning among sub-units	Fully aligned	Principle-based (pressure cascade zoning logic)	Sub-units with differing risk levels are addressed within separate pressure zones	Number and boundaries of zones vary with laboratory scale	Segregation of grossing, technical areas, offices, and support spaces	“Pressure gradation according to functional zones”	NRC; DiBerardinis et al.
Doors, transitions, and pressure continuity	Partially aligned	Principle-based (pressure continuity through transitional design)	Doors are planned to avoid disruption of pressure continuity	Interpretation of automatic doors, double doors, and airlocks varies	Door type selection; need for airlocks; circulation layout	Recommendation for airlocks, particularly in high-risk areas	CLSI; NHS
HVAC control and monitoring	Partially aligned	Principle-based (traceable pressure control logic)	Pressure regimes are expected to be traceable and controllable	Continuous monitoring versus periodic inspection approaches	Sensor placement; alarm systems; monitoring infrastructure	Continuous differential pressure monitoring (principle-based)	ASHRAE; UNC
Continuity under failure scenarios	Fully aligned	Principle-based (fail-safe containment logic)	Pressure regimes should remain in a safe state during system failures	Treated as a design issue versus an operational issue	Redundant systems; fan placement; emergency operation scenarios	“Safe-state under failure mode” approach	NHS; ASHRAE
Interaction with human behavior	Fully aligned	Principle-based (engineering-over-behavior safety hierarchy)	Pressure regimes are intended to function independently of user behavior	Degree of reliance on training and user compliance varies	Level of automation; limitation of user intervention	Priority for automated control (no numerical threshold)	NHS; NRC

**HVAC:** Heating, Ventilation, and Air Conditioning **Note:** Alignment categories were assigned according to the predefined analytical framework described in the Methods section. Classification reflects cross-document consistency in containment principles and implementation specificity

In this context, the pressure regime should be considered not merely as a technical output of the ventilation system, but as a comprehensive planning parameter that directly affects the PLT plan type, the clean-contaminated area separation, and infrastructure continuity (12,14).

### Chemical Vapor Control and Local Exhaust Solutions

Comparative analysis identifies chemical vapor control as a core infrastructure parameter in pathology laboratories due to routine exposure to volatile substances such as formaldehyde and xylene. Across reviewed guidelines, effective control is consistently linked to source-based capture rather than reliance on general ventilation alone (Table 7) (2,4,7,10).

**Table 7:** Chemical Vapor Control and Local Exhaust Solutions (Fume Hoods): A Comparative Analysis

**Evaluated Dimension**	**Alignment Category**	**Operational Basis**	**Common Approach in Guidelines / Standards**	**Diverging Approaches**	**Impact on Planning Decisions**	**Example of Numerical Threshold / Technical Criterion**	**Source Mapping**
Source control principle	Fully aligned	Principle-based	Vapor capture at source prioritized over dilution	General ventilation vs mandatory local exhaust	LEV required in grossing/chemical areas	No numeric threshold (hierarchy-of-controls)	OSHA; CLSI; NHS; NRC
Fume hood / cabinet placement	Partially aligned	Mixed (principle-based + selective numeric spacing guidance)	Avoid turbulence and cross-drafts	Clearance distances and layout rules vary	Spatial buffer; diffuser coordination	≥1 m clearance from the fume hood face to the nearest obstruction/traffic route	MEE; DiBerardinis; NHS; NERC
Supply air-exhaust interaction	Partially aligned	Mixed (performance-based testing + layout guidance)	Supply air must not impair hood capture	Performance testing vs prescriptive layout	Diffuser positioning; jet velocity control	Avoid excessive face velocities	DiBerardinis; UNC
Heavier-than-air vapors and down-draught	Partially aligned	Principle-based (chemical property-dependent capture logic)	Near-floor capture for dense vapors	Down-draught vs general exhaust reliance	Low-level extraction design	Down-draught / near-floor exhaust	NERC; NHS; iHFG
Emergency exhaust capacity	Partially aligned	Scenario-based (emergency/purge ventilation mode)	Rapid exhaust response during chemical incidents is required	Manual vs automated activation	Emergency mode integration	Target of ~12 ACH under emergency conditions	UNC
Safe discharge of exhaust air	Fully aligned	Mixed (distance-based guidance + performance-based dispersion modeling)	Exhaust air discharged away from intakes and occupied areas	Distance vs dispersion modeling	Roof discharge height, direction, velocity, and building geometry	≥3 m vertical discharge above roof level	ASHRAE (2019); ASHRAE (2021)
Measurement, monitoring, and occupational safety	Partially aligned	Regulatory threshold-based (exposure limit frameworks)	Exposure management within occupational safety frameworks	Level of detail in monitoring guidance varies	Defined sampling and responsibility	Principle-based (limit values set by regulatory bodies)	OSHA (2011a, 2011b)

**ACH:** Air Changes per Hour, **LEV: **Local Exhaust Ventilation **Note:** Alignment categories were assigned according to the predefined analytical framework described in the Methods section. Numerical examples reflect guidance values reported in the referenced documents and may be context-dependent.

Local exhaust systems are defined not merely by their presence, but by capture efficiency, proximity to the emission source, and integration with user workflow (14,15,21). Several documents emphasize that airflow direction and immediate vapor removal prior to entry into the breathing zone are more critical than fixed air velocity values.

For heavy vapors, including formaldehyde and xylene, ceiling-mounted exhaust systems are considered insufficient. Counter-level or near-floor “down-draught” configurations are described as more effective in preventing vapor accumulation in the breathing zone (7,14).

Divergence among guidelines concerns the degree of prescriptive specification. Some define measurable criteria, including hood opening dimensions and face velocities, whereas others frame local exhaust performance within broader ventilation and pressure-regime integration (4,10,19) .

Collectively, the findings indicate that local exhaust systems function as integrated infrastructure components coordinated with ventilation strategy and spatial configuration, rather than isolated equipment installations.

### Floor, Wall, and Surface Materials

Floor, wall, and surface materials in PLs should be addressed not merely as elements of spatial aesthetics or hygiene, but as critical planning inputs directly linked to chemical exposure control, biological contamination management, occupational safety, and infrastructure continuity (Table 8). Comparative analysis of international guidelines reveals a strong consensus that surface material selection must be driven by anticipated chemical load, mechanical stress, and cleaning intensity rather than generic healthcare standards (2,7,9).

**Table 8:** Floor, Wall, and Surface Materials in Pathology Laboratories: A Comparative Analysis

**Evaluated Dimension**	**Alignment Category**	**Operational Basis**	**Common Approach in Guidelines / Standards**	**Diverging Approaches**	**Impact on Planning Decisions**	**Example of Numerical Threshold / Technical Criterion**	**Source Mapping**
Chemical resistance of floor surfaces	Fully aligned	Principle-based (hygiene and chemical resistance logic)	Non-porous, chemically resistant flooring required	Material-specific vs performance-based	Selection of epoxy/vinyl systems	Liquid-impermeable surface	NRC; NHS
Joints and seam design	Fully aligned	Principle-based (contamination control and cleanability logic)	Minimization of joints and secure sealing are emphasized to reduce contamination risk	Modular panels vs general sealing	Large-format flooring installation; heat-welded seams	Reduction in number of joints	iHFG
Wall-floor junctions	Partially aligned	Principle-based (infection control detailing logic)	Curved (coved) junctions are recommended to support contamination control and cleanability	Treated as a requirement in some guidelines and as a preference in others	Early resolution of wall-floor detailing during design phase	Use of coved junctions instead of sharp corners	NHS; NRC
Cleanability of walls and vertical surfaces	Fully aligned	Principle-based (cleanability and chemical durability logic)	Smooth, washable, and chemically resistant wall surfaces are recommended	Balance between aesthetic finishes and technical coatings varies	Risk-based selection of wall paint and coating systems	Chemically resistant coating (principle-based)	CLSI
Functional impact of surface color and finish	Partially aligned	Principle-based (visual ergonomics and inspection support logic)	Light-colored, non-glare surfaces are accepted to support visual comfort and inspection	Neutral stance prioritizing performance over color selection	Differentiated color and finish strategies by unit type	Matte, non-reflective finish (principle-based)	CLSI
Compatibility with maintenance and renovation processes	Partially aligned	Principle-based (life-cycle and serviceability logic)	Surface systems are expected to support local repair and ease of intervention	Addressed as a design principle versus an operational concern	Preference for modular and demountable surface systems	Surface systems allowing localized repair (principle-based)	NRC; iHFG

**Note:** Alignment categories reflect cross-document consistency in surface performance and detailing requirements. Numerical thresholds are generally not specified; guidance is predominantly principle-based.

For flooring systems, impermeability, chemical resistance, and long-term durability emerge as core criteria distinguishing PLs from administrative or office environments. Materials susceptible to cracking, porosity, or excessive jointing are consistently discouraged, as they increase contamination risk, complicate decontamination procedures, and elevate long-term maintenance demands. Consequently, guidelines converge on the use of large-format, seamless floor coverings with hot-welded joints and coved wall transitions to ensure leak-proof continuity (7,10).

For walls and vertical surfaces, non-absorbent, smooth, and chemically resistant finishes are recommended, particularly in splash-prone or high-cleaning areas. Sealed junctions and rounded transitions are repeatedly emphasized to reduce contamination retention and facilitate effective decontamination (2,12).

Across documents, material selection is evaluated in terms of long-term performance and maintenance stability. Surfaces lacking chemical and mechanical resilience are associated with premature renewal and operational disruption, positioning surface specification as an infrastructure-level planning parameter rather than a purely architectural decision (7,12).

### Emergency Eye Wash Stations and Safety Equipment

Comparative analysis identifies emergency eye wash stations as a mandatory component of chemical risk management in pathology laboratories (Table 9). Strong consensus exists regarding their required presence; however, guidelines differ in spatial integration and operational criteria. Some documents specify numerical thresholds for access distance, response time, and system continuity, whereas others leave implementation details to institutional planning authority (10,14,16,21–23).

**Table 9:** Emergency Equipment and Safety Infrastructure in Pathology Laboratories: A Comparative Analysis

**Evaluated Dimension**	**Alignment Category**	**Operational Basis**	**Common Approach in Guidelines / Standards**	**Diverging Approaches**	**Example of Numerical Threshold / Technical Criterion**	**Impact on Planning Decisions**	**Source Mapping**
Emergency showers and eyewash stations	Fully aligned	Principle-based (rapid drenching capability)	Safety showers required where corrosive exposure risk exists	Level of planning detail varies	Immediate drenching capability	Plumbing integration; wet-area design; access clearance	OSHA; NRC; NHS
Distribution adequacy	Partially aligned	Principle-based (risk-based distribution)	Number and placement based on hazard profile	“Adequate” defined variably	Risk-based allocation	Zonal risk mapping; renovation impact	OSHA; NRC; CLSI
Spill response infrastructure	Partially aligned	Principle-based (incident readiness)	Spill kits and absorbents available in hazard areas	Emphasis on engineering vs procedural control differs	No uniform numeric threshold	Storage location planning; accessibility	NRC; OSHA
Fire response equipment	Partially aligned	Principle-based (first-response readiness)	Fire extinguishers available near hazard zones	Type and positioning governed by local code	Readily available equipment	Corridor/interface planning; inspection routes	NRC; NHS
Exposure monitoring governance	Partially aligned	Regulatory threshold-based (exposure limit framework)	Chemical exposure managed within regulatory limits	Monitoring frequency and method vary	PEL-based limits (chemical-specific)	Definition of monitoring points and responsibilities	OSHA; CLSI

**PEL**: Permissible Exposure Limit **Note:** Alignment reflects cross-document consistency in safety equipment requirements. Most criteria are principle-based; exposure limits are defined by regulatory authorities

All reviewed sources agree that portable or faucet-mounted units cannot replace fixed emergency stations and should be considered supplementary measures (14,15). Infrastructure requirements-such as independent water supply, controlled temperature range, adequate flow rate, and drainage capacity-are repeatedly emphasized as necessary for functional reliability, linking emergency equipment to plumbing design and maintenance accessibility (10,23).

These findings demonstrate cross-document agreement on safety intent, with variation primarily at the level of implementation detail rather than principle.

### Information Technology and Digital Pathology Infrastructure

Comparative analysis indicates that information technology infrastructure in pathology laboratories directly influences space allocation, energy supply, and environmental control requirements (Table 10). Guidelines consistently address laboratory information systems (LIS), digital pathology platforms, and remote access solutions as infrastructure components with spatial and technical implications (5,6,10,12).

**Table 10:** Information Technologies and Digital Pathology Infrastructure in Pathology Laboratories: A Comparative Analysis

**Evaluated Dimension**	**Alignment Category**	**Operational Basis**	**Common Approach in Guidelines / Standards**	**Diverging Approaches**	**Impact on Planning Decisions**	**Example of Numerical Threshold / Technical Criterion**	**Source Mapping**
End-to-end digital pathology system	Partially aligned	System-architecture based	WSI integrated with IMS, LIS, and viewing platforms	Scanner-centered definitions versus system-architecture-oriented approaches	IMS/LIS integration; archive topology; workstation standardization	Component-based system definition (no numerical threshold)	FDA
Storage and archive architecture	Partially aligned	Performance-based (data integrity & retention)	Scalable storage with long-term accessibility	Local vs centralized; proprietary vs standards-based	Server and storage capacity planning; backup strategies; data migration	Long-term retention principle	NHS; iHFG; DICOM
Interoperability and standards	Partially aligned	Performance-based	Emphasis on data standardization and portability across systems	DICOM-based vs vendor-locked systems	Integration cost; vendor lock-in risk	DICOM WSI framework (Supplement 145)	DICOM
Physical footprint of IT infrastructure	Partially aligned	Performance-based (diagnostic usability)	Space, power supply, and cooling loads are incorporated into infrastructure planning	General IT guidance vs detailed specs	Server rooms; UPS; cooling; maintenance access	Business continuity-oriented infrastructure approach	NHS; iHFG
Continuity and failure scenarios	Fully aligned	Regulatory-based (clinical validation framework	Diagnostic output continuity requires redundancy and planned maintenance	Degree of redundancy and monitoring varies by guideline	Redundant networks and storage; maintenance windows; secure update strategies	Continuity principle (no numerical threshold)	NHS; iHFG; FDA

**DICOM: **Digital Imaging and Communications in Medicine, **IMS: **Image Management System, **IT:** Information Technology, **LIS:** Laboratory Information System, **UPS:** Uninterruptible Power Supply, **WSI:** Whole Slide Imaging **Note:** Digital pathology infrastructure criteria are predominantly performance-based and system-architecture oriented. Alignment categories reflect variability in prescriptive detail rather than absence of conceptual consensus.

Integration of digital pathology into clinical workflows is defined through standardized validation and quality assurance frameworks rather than isolated equipment deployment (24–28). This positioning links digital systems to operational reliability and workflow continuity.

Slide scanners, high-resolution imaging systems, and large-volume data storage introduce additional infrastructure demands, including network capacity, uninterruptible power supply (UPS), backup systems, cooling requirements associated with increased heat load, and accessible maintenance zones (6,10,12,25,26,28–30).

Across the reviewed documents, digital infrastructure is evaluated as a long-term operational parameter requiring scalability and continuity planning, rather than as an auxiliary technological addition (10,12,27).

### Human-Infrastructure Interaction

Comparative findings indicate that lighting, noise, spatial dimensions, and circulation are frequently addressed as separate technical parameters. However, cross-document evaluation demonstrates that these variables operate in combination, influencing workflow stability and staff interaction patterns within the laboratory environment (Table 11).

**Table 11:** Human-Infrastructure Interaction Parameters in Pathology Laboratories - Integrated Comparative Analysis

**Human-Infrastructure Parameter**	**Alignment Category**	**Operational Basis**	**Common Approach**	**Diverging Approaches**	**Impact on Planning Decisions**	**Example Criterion**	**Source Mapping**
Lighting level and task illumination	Partially aligned	Performance-based	Higher-than-office illumination; task lighting required	Fixed lux vs task-adjusted ranges	Combined design of general and adjustable task lighting in diagnostic areas	500-750 lux (technical areas)	NHS HBN 15; iHFG; CLSI
Color rendering and visual perception	Partially aligned	Performance-based	High CRI is preferred where color discrimination is critical	Minimum-threshold (CRI ≥80) vs. higher-quality (CRI ≥90) approaches	Luminaire selection becomes a diagnostic reliability parameter	CRI ≥80 (minimum), preferably ≥90	CIE; CLSI
Glare, shadows, and daylight control	Partially aligned	Performance-based	Avoidance of glare and harsh shadows is a shared principle	Inclusion of glare metrics vs. qualitative guidance	Use of indirect lighting, diffuse luminaires, controlled daylight.	UGR referenced in some guidelines; no fixed threshold	NHS; DiBerardinis et al.
Mechanical noise sources	Partially aligned	Occupational health-based (exposure control logic)	HVAC and mechanical systems are major contributors to background noise	Design-principle-based vs. dB-defined approaches	Equipment selection and spatial separation of mechanical rooms	≤85 dB(A) for long-term exposure (legal limit)	OSHA; ASHRAE
Spatial and acoustic zoning	Partially aligned	Design-based (environmental control and concentration logic)	Source control and spatial segregation are recommended	Priority given to acoustic design varies across guidelines	Functional zoning of high-concentration work areas	Principle-based; no numerical threshold	FGI; NRC
Corridor width	Partially aligned	Function-based (circulation safety and equipment logistics logic)	Corridors must safely support personnel, equipment, and specimen movement	Prescriptive minimum widths vs. function-based adequacy	Corridor cross-sections designed according to equipment dimensions	Functional clearance principle	NRC; Singh et al.
Organizational circulation	Partially aligned	Behavior-based (workflow and organizational efficiency logic)	Circulation areas influence user behavior and organizational culture	Technical vs. behavioral framing differs	Avoidance of corridors becoming storage or conflict zones	Conceptual framework; no numerical threshold	Skolozdra
Thermal comfort	Partially aligned	Performance-based	Temperature and humidity affect user comfort and continuity	Treated as comfort vs. performance parameter	User-centered HVAC zoning in long-duration work areas	Summer: 22-26 °C; Winter: 20-24 °C	CLSI; NRC
Relative humidity stability	Partially aligned	Performance-based	Stable humidity supports comfort and attention	Equipment-focused vs. human-centered approaches	Continuous monitoring and control of humidity	30-60% RH (preferably 40-60%)	CLSI; UNC
User density and system responsiveness	Partially aligned	Performance-based	HVAC adapts to variable occupancy	Fixed setpoints vs. dynamic control approaches	HVAC systems designed for variable loads and adaptability	Dynamic control principle	NHS; UNC

**ACH**: air changes per hour, **CIE:** International Commission on Illumination, **CRI**: Color Rendering Index, **HVAC:** Heating, Ventilation, and Air Conditioning, **RH**: Relative Humidity, **UGR:** Unified Glare Rating. **Note:** Human-infrastructure interaction parameters are predominantly performance-based and ergonomics-oriented. Alignment categories reflect variability in prescriptive thresholds rather than absence of conceptual consensus across guidelines.

Spatial configuration and zoning are associated with variations in inter-unit accessibility and operational coordination. Excessive compartmentalization, extended circulation routes, or acoustically disruptive environments are linked to reduced interaction efficiency, whereas controlled separation with maintained accessibility supports coordinated workflow organization (2,5,7,10).

### Illumination Level and Color Rendering

In areas requiring precise color discrimination-such as macroscopy, microtomy, and microscopy-color rendering is defined as a critical complement to illumination intensity (Table 11). While general laboratory lighting levels are specified in lux values, higher color rendering performance is recommended for pathology-specific tasks (5,10,12,15).

Lighting standards commonly define CRI ≥80 as a minimum threshold; however, documents addressing diagnostic environments reference a preference for CRI ≥90 where color perception directly affects interpretation accuracy (31,32). This differentiation is framed as a performance-oriented planning consideration rather than a universal mandatory requirement (15,33).

Guidelines further indicate that uniform lighting alone is insufficient. Integration of general and task lighting, glare control, shadow reduction, and avoidance of color distortion are consistently emphasized. Natural light may be incorporated selectively; however, uncontrolled direct sunlight is discouraged due to potential color distortion and visual adaptation effects (7,10,12,34).

### Noise Level

Noise is addressed as an environmental infrastructure parameter affecting attention during cognitively demanding diagnostic tasks (Table 11). Experimental ergonomics studies demonstrate that background noise can reduce concentration and cognitive performance in sustained attention tasks (10,24,35,36).

Regulatory thresholds, including the 85 dB(A) eight-hour exposure limit defined by OSHA and EU legislation, focus on hearing protection rather than optimal cognitive performance (23,37).

Guidelines evaluate noise management within infrastructure planning, emphasizing mechanical equipment placement, acoustic zoning, and sound-absorptive materials (13,17). Spatial separation of high-concentration work areas from mechanical sources and source-level noise reduction are consistently identified as planning measures.

### Corridor Widths and Circulation Areas

Corridors function as operational infrastructure elements affecting workflow continuity and safety (Table 11) (2,10). Comparative findings indicate that insufficient corridor width restricts personnel movement and equipment transfer, while excessive width reduces spatial efficiency and alters usage behavior (1,14).

Planning approaches prioritize functional adequacy rather than maximum dimensional standards. Circulation routes are recommended to accommodate maintenance, equipment renewal, and emergency evacuation scenarios (2,10). Routing service distribution lines through corridors is identified as a continuity strategy enabling infrastructure intervention without disrupting laboratory operations (2,10,38).

### Temperature and Humidity Conditions

Temperature and humidity are addressed as environmental parameters influencing staff performance and operational stability (Table 11) (14,15). Guidelines evaluate these conditions in relation to both equipment reliability and human comfort (7,18).

In microscopy and prolonged standing tasks, impaired thermal comfort is associated with reduced concentration and increased error risk (7,14,15). Accordingly, documents emphasize stable maintenance of recommended ranges throughout operational hours (15,18).

Temperature and humidity are framed as dynamic parameters influenced by workload, equipment heat load, spatial organization, and user density (2,5,7,10,15,18). This positioning integrates thermal control within broader infrastructure planning rather than treating it as an isolated HVAC output.

## DISCUSSION

The findings demonstrate that physical infrastructure requirements for pathology laboratories cannot be reduced to uniform numerical thresholds or single implementation models. Variability across guidelines reflects differences in risk profiles, workflow structures, and institutional contexts inherent to PL practice (2,10,12). Divergence among documents therefore represents contextual adaptation rather than conceptual inconsistency.

In ventilation-related parameters-particularly air change rates, pressure regimes, and airflow direction-prescriptive thresholds remain influential. However, the results indicate that numerical values alone do not determine safety. Ventilation performance depends on coordinated air management, including airflow directionality, pressure zoning, and local exhaust integration. Elevated ACH values without appropriate diffuser configuration may compromise containment performance (4,7,11,18).

Similarly, chemical vapor control findings confirm that fume hoods and local exhaust systems function as primary control mechanisms independent of central ventilation rates. Their effectiveness is determined by capture efficiency, spatial positioning, and system integration within ventilation and pressure regimes (2,7,10). This underscores the importance of evaluating local exhaust as an infrastructure system rather than as isolated equipment.

Environmental parameters-including lighting and noise-emerged as determinants of diagnostic working conditions. While guidelines define minimum thresholds, sustained-attention tasks in pathology practice require planning strategies that address concentration demands beyond baseline compliance levels (2,15,22). Infrastructure decisions therefore influence not only safety but also diagnostic stability.

Surface material findings extend continuity considerations beyond mechanical systems. Chemical resistance, cleanability, and maintenance feasibility were consistently linked to long-term operational reliability, positioning material selection within lifecycle infrastructure planning (2,7,12).

Regarding digital pathology, the analysis indicates that digital systems introduce physical infrastructure implications involving energy capacity, cooling, network architecture, and spatial allocation. Digital integration thus represents an infrastructural planning requirement rather than solely a technological implementation issue (28–30).

Collectively, these findings indicate that pathology laboratory planning requires translation of international standards into context-sensitive infrastructure strategies. Rather than functioning as fixed design prescriptions, the reviewed guidelines operate as reference frameworks that must be interpreted in relation to institutional constraints, operational risk profiles, and laboratory workflow structures. Within this analytical perspective, infrastructure parameters emerge as interdependent planning variables whose effectiveness depends on coordinated integration during laboratory establishment and renewal processes (2,10,12).

## CONCLUSION

Across the 38 normative and technical documents included in the analytical dataset, no parameter domain demonstrated direct contradiction in core safety principles. Observed variation occurred primarily at the level of operational specification and threshold articulation rather than conceptual disagreement. Strong cross-document alignment was identified in pressure containment strategies, directional airflow control, and source-based vapor capture, indicating stable consensus in infrastructure parameters directly related to exposure control.

In contrast, greater variability emerged in domains governed by quantitative thresholds, particularly air change rates (ACH), illumination parameters, and selected environmental comfort indicators. Divergent implementation patterns were primarily associated with differences between prescriptive and performance-based approaches, as well as between static infrastructure models and adaptive planning frameworks designed to accommodate technological change and operational continuity.

Parameters related to human-infrastructure interaction, including acoustic zoning, corridor configuration, and thermal comfort conditions, were more frequently addressed through qualitative or principle-based guidance rather than standardized numeric criteria. In these domains, interpretative responsibility shifts toward planners and institutional risk assessments, particularly in renovation scenarios where spatial constraints and operational continuity requirements must be balanced.

Overall, the comparative synthesis indicates that high-risk containment parameters demonstrate strong cross-document consensus, whereas adaptability-sensitive infrastructure domains exhibit structured variability. These findings suggest that physical infrastructure planning in pathology laboratories should rely on risk-informed interpretation of international guidance rather than direct transfer of prescriptive thresholds, supporting flexible and context-responsive planning strategies in both new-build and renovation settings.

## Conflict of Interest

The author declares that there is no conflict of interest regarding the topics covered in this study.

## Funding

This research did not receive any specific grant from funding agencies in the public, commercial, or not-for-profit sectors.

## Declaration of generative AI use

During the preparation of this study, the author used ChatGPT to create comparative tables and improve their language and readability. After using this tool/service, the author reviewed and edited the content as needed and is fully responsible for the content of the published article.

## Supplementary Material

**Supplementary Table SI:** Exploratory Document Mapping Results

File_Name	File_Type	Exclusion_Code	Exclusion_Reason
6324723e1b1f8 (1).docx	.docx	E1	Did not meet predefined operational infrastructure criteria upon full-text review
Laboratory Design; Approved Guideline, second edition (2).docx	.docx	E2	General technical/educational reference without direct operational infrastructure thresholds
The American Institute of Architects Academy of Architecture for Health.docx	.docx	E1	Did not meet predefined operational infrastructure criteria upon full-text review
moleculargeneticpathologysupplementalguidetemplate.docx	.docx	E2	General technical/educational reference without direct operational infrastructure thresholds
went.docx	.docx	E1	Did not meet predefined operational infrastructure criteria upon full-text review
1,2,3,4.pdf	.pdf	E1	Did not meet predefined operational infrastructure criteria upon full-text review
2008_0925_Mercer_Lab-Space-Planning-and-Facilities-Design_Lab-Quality-Confab.pdf	.pdf	E2	General technical/educational reference without direct operational infrastructure thresholds
9789240011311-eng.pdf	.pdf	E1	Did not meet predefined operational infrastructure criteria upon full-text review
APJ_Mesl_Risk.pdf	.pdf	E1	Did not meet predefined operational infrastructure criteria upon full-text review
ASHEIR 2018 pat lab ventilasyon bilgileri.pdf	.pdf	E1	Did not meet predefined operational infrastructure criteria upon full-text review
Bancroft’s Theory and Practice of Histological Techniques (9. basım).pdf	.pdf	E1	Did not meet predefined operational infrastructure criteria upon full-text review
Basic and Advanced laboratory Techniques in Histopathology and Cytology.pdf	.pdf	E3	Peer-reviewed/academic content not primarily focused on physical infrastructure criteria
Biyogüvenlik Laboratuvar Seviyeleri ve biyogüvenlik kabinlerin seçim ve bakımı.pdf	.pdf	E1	Did not meet predefined operational infrastructure criteria upon full-text review
Document1.pdf	.pdf	E1	Did not meet predefined operational infrastructure criteria upon full-text review
GP18-A2 Laboratory design.pdf	.pdf	E2	General technical/educational reference without direct operational infrastructure thresholds
Improving Histopathology Laboratory Productivity.pdf	.pdf	E3	Peer-reviewed/academic content not primarily focused on physical infrastructure criteria
Koss Diagnostic Cytology and Its Histopathologic Bases 5th Ed [PDF][tahir99] VRG (1).pdf	.pdf	E3	Peer-reviewed/academic content not primarily focused on physical infrastructure criteria
Laboratory-Design-Guidelines.pdf	.pdf	E2	General technical/educational reference without direct operational infrastructure thresholds
Laboratory-Ventilation-Codes-and-Standards--Application-Guide_A6V10324363_us-en.pdf	.pdf	E2	General technical/educational reference without direct operational infrastructure thresholds
Lean Hospitals_ Improving Quality, Patient Safety, and Employee Engagement, Second Edition.pdf	.pdf	E1	Did not meet predefined operational infrastructure criteria upon full-text review
MODERN PATHOLOGY.pdf	.pdf	E3	Peer-reviewed/academic content not primarily focused on physical infrastructure criteria
Molecular Diagnostics in Clinical Oncology.pdf	.pdf	E1	Did not meet predefined operational infrastructure criteria upon full-text review
Molecular pathology e The value of an integrative approach-AAA+.pdf	.pdf	E3	Peer-reviewed/academic content not primarily focused on physical infrastructure criteria
Molecular pathology e The value of an integrative approach.pdf	.pdf	E3	Peer-reviewed/academic content not primarily focused on physical infrastructure criteria
Pathology Archive- Evaluation of Integrity, Regulatory Compliance, and Construction of Searchable Database From Print Reports.pdf	.pdf	E2	General technical/educational reference without direct operational infrastructure thresholds
Patoloji Pratiğinde Güncel Değişim- Akılcı Laboratuvar Uygulaması.pdf	.pdf	E1	Did not meet predefined operational infrastructure criteria upon full-text review
Patoloji laboratuarında mesleki riskler ve güvenlik önlemleri.pdf	.pdf	E1	Did not meet predefined operational infrastructure criteria upon full-text review
SETLAB ÇEKER OCAK TEKNİK ŞARTNAME.pdf	.pdf	E1	Did not meet predefined operational infrastructure criteria upon full-text review
SPECIFIC CRITERIA for ACCREDITATION OF MEDICAL LABORATORIES (personel sayı ve niteliği açık).pdf	.pdf	E1	Did not meet predefined operational infrastructure criteria upon full-text review
Translational AI and Deep Learning in Diagnostic Pathology.pdf	.pdf	E3	Peer-reviewed/academic content not primarily focused on physical infrastructure criteria
Using six sigma in clinical laboratory.pdf	.pdf	E1	Did not meet predefined operational infrastructure criteria upon full-text review
hindistan sağlık bakanlığı Guideline doc design of BSL2 labs(dist,hosp,chc&phc) level.pdf	.pdf	E2	General technical/educational reference without direct operational infrastructure thresholds
6.pdf	.pdf	E1	Did not meet predefined operational infrastructure criteria upon full-text review
laboratory-design-guide 3. edition.pdf	.pdf	E2	General technical/educational reference without direct operational infrastructure thresholds
molecular pathology standard checklist.pdf	.pdf	E1	Did not meet predefined operational infrastructure criteria upon full-text review
practical-guide-specimen-handling.pdf	.pdf	E2	General technical/educational reference without direct operational infrastructure thresholds
7.txt	.txt	E1	Did not meet predefined operational infrastructure criteria upon full-text review
GermlineValidationTables.xlsx	.xlsx	E3	Peer-reviewed/academic content not primarily focused on physical infrastructure criteria

E1 - Did not meet predefined operational infrastructure criteria upon full-text review

E2 - General technical/educational reference without direct operational infrastructure thresholds

E3 - Peer-reviewed/academic content not primarily focused on physical infrastructure criteria

**Supplementary Table SII:** Excluded Documents and Reasons for Exclusion

Domain	Institutional Type	Infrastructure Theme	Role in Study	Supporting Files	URLs Extracted	Example URL
cdc.gov	Governmental body	Biosafety / chemical safety	Exploratory parameter framing only	8	8	http://www.cdc.gov/
rcpath.org	Professional / standards organization	Laboratory design / general infrastructure	Exploratory parameter framing only	5	3	http://www.rcpath.org/publications-media/publications/datasets/datasets-TP
osha.gov	Governmental body	Biosafety / chemical safety	Exploratory parameter framing only	4	4	http://www.osha.gov/
cap.org	Professional / standards organization	Laboratory design / general infrastructure	Exploratory parameter framing only	3	5	http://www.cap.org/
fda.gov	Governmental body	Laboratory design / general infrastructure	Exploratory parameter framing only	3	5	http://www.fda.gov/MedicalDevices/DeviceReg
nfpa.org	Professional / standards organization	Laboratory design / general infrastructure	Exploratory parameter framing only	3	1	http://www.nfpa.org/
pathology.nl	Other / mixed	Laboratory design / general infrastructure	Exploratory parameter framing only	3	1	http://www.pathology.nl
healthfacilityguidelines.com.au	Australian institution	Laboratory design / general infrastructure	Exploratory parameter framing only	2	2	https://healthfacilityguidelines.com.au/health
nap.edu	Academic institution	Laboratory design / general infrastructure	Exploratory parameter framing only	2	2	http://www.nap.edu
ncjrs.org	Professional / standards organization	Laboratory design / general infrastructure	Exploratory parameter framing only	2	2	http://www.ncjrs.org/txtfiles/168106.txt
ansi.org	Professional / standards organization	Laboratory design / general infrastructure	Exploratory parameter framing only	2	1	http://www.ansi.org/
ashrae.org	Professional / standards organization	HVAC / ventilation	Exploratory parameter framing only	2	1	http://www.ashrae.org/
clsi.org	Professional / standards organization	Laboratory design / general infrastructure	Exploratory parameter framing only	2	1	http://www.clsi.org/
clsi.org	Professional / standards organization	Laboratory design / general infrastructure	Exploratory parameter framing only	2	1	http://www.clsi.org./
dergipark.org.tr	Other / mixed	Laboratory design / general infrastructure	Exploratory parameter framing only	2	1	https://dergipark.org.tr/tr/pub/inonusaglik/writing-rules
hc-sc.gc.ca	Other / mixed	Biosafety / chemical safety	Exploratory parameter framing only	2	1	http://www.hc-sc.gc.ca/hpb/lcdc/biosafty/docs/index.html
hhs.gov	Governmental body	Laboratory design / general infrastructure	Exploratory parameter framing only	2	1	http://www.hhs.gov/
iccsafe.org	Professional / standards organization	Laboratory design / general infrastructure	Exploratory parameter framing only	2	1	http://www.iccsafe.org/
iesna.org	Professional / standards organization	Laboratory design / general infrastructure	Exploratory parameter framing only	2	1	http://www.iesna.org/
iest.org	Professional / standards organization	Laboratory design / general infrastructure	Exploratory parameter framing only	2	1	http://www.iest.org/
iso.org	Professional / standards organization	Laboratory design / general infrastructure	Exploratory parameter framing only	2	1	https://www.iso.org/standard/39883.html
jcaho.org	Professional / standards organization	Laboratory design / general infrastructure	Exploratory parameter framing only	2	1	http://www.jcaho.org/
lrc.rpi.edu	Academic institution	Laboratory design / general infrastructure	Exploratory parameter framing only	2	1	http://www.lrc.rpi.edu/
niehs.nih.gov	Governmental body	Laboratory design / general infrastructure	Exploratory parameter framing only	2	1	http://niehs.nih.gov/obhsb/ergoguid/home.htm
nih.gov	Governmental body	Laboratory design / general infrastructure	Exploratory parameter framing only	2	1	http://www.nih.gov/
sefalabs.com	Other / mixed	Laboratory design / general infrastructure	Exploratory parameter framing only	2	1	http://www.sefalabs.com/
soft-tox.org	Professional / standards organization	Laboratory design / general infrastructure	Exploratory parameter framing only	2	1	http://www.soft-tox.org/
usdoj.gov	Governmental body	Laboratory design / general infrastructure	Exploratory parameter framing only	2	1	http://www.usdoj.gov/crt/ada/taprog.htm
who.int	International organization	Biosafety / chemical safety	Exploratory parameter framing only	2	1	http://www.who.int/about/licensing/copyright_form/en/index.html
acgme.org	Professional / standards organization	Lighting	Exploratory parameter framing only	1	12	https://www.acgme.org/Meetings-and-Educational-Activities/Other-Educational-Activities/Courses-and-Workshops/Developing-Faculty-Competencies-in-Assessment
hmso.gov.uk	Governmental body	Laboratory design / general infrastructure	Exploratory parameter framing only	1	8	http://www.hmso.gov.uk/si/si1989/Uksi_19891790_en_1.htm
www-ncbi-nlm-nih-gov.ezproxy.libraries.wright.edu	Academic institution	Laboratory design / general infrastructure	Exploratory parameter framing only	1	6	https://www-ncbi-nlm-nih-gov.ezproxy.libraries.wright.edu/pubmed/?term=Gonnella%20JS%5BAuthor%5D&cauthor=true&cauthor_uid=19638773
dir.ca.gov	Governmental body	Laboratory design / general infrastructure	Exploratory parameter framing only	1	4	http://www.dir.ca.gov/
nerc.ac.uk	UK public/academic institution	Laboratory design / general infrastructure	Exploratory parameter framing only	1	4	http://www.nerc.ac.uk/about/polic
ahrq.gov	Governmental body	Laboratory design / general infrastructure	Exploratory parameter framing only	1	2	https://www.ahrq.gov/talkingquality/measures/setting/physician/index.html
amp.org	Professional / standards organization	Laboratory design / general infrastructure	Exploratory parameter framing only	1	2	https://www.amp.org/education/emerging-and-evolving-biomarkers/
datacenter.commonwealthfund.org	Professional / standards organization	Laboratory design / general infrastructure	Exploratory parameter framing only	1	2	https://datacenter.commonwealthfund.org/
dl.acgme.org	Professional / standards organization	Laboratory design / general infrastructure	Exploratory parameter framing only	1	2	https://dl.acgme.org/
haad.ae	Other / mixed	Laboratory design / general infrastructure	Exploratory parameter framing only	1	2	https://www.haad.ae/HAAD/
acs.org	Professional / standards organization	Laboratory design / general infrastructure	Exploratory parameter framing only	1	1	http://acs.org/safety
aiha.org	Professional / standards organization	Laboratory design / general infrastructure	Exploratory parameter framing only	1	1	http://www.aiha.org/
bgsm.edu	Academic institution	Laboratory design / general infrastructure	Exploratory parameter framing only	1	1	http://www.bgsm.edu/ehs/sp_occ_laberg.html
clsi.org	Professional / standards organization	Lighting	Exploratory parameter framing only	1	1	https://clsi.org/about/blog/efficient-medical-laboratory-planning-and-design/
dmat.abudhabi.ae	Other / mixed	Laboratory design / general infrastructure	Exploratory parameter framing only	1	1	https://dmat.abudhabi.ae/en/About/Pages/buildingcode.aspx
dosyamerkez.saglik.gov.tr	Governmental body	Laboratory design / general infrastructure	Exploratory parameter framing only	1	1	https://dosyamerkez.saglik.gov.tr/Eklenti/25054,akilci-
ehs.ucr.edu	Academic institution	Laboratory design / general infrastructure	Exploratory parameter framing only	1	1	http://ehs.ucr.edu/ehsacademy/presentations/ergonomicslaboratorychecklist.pdf
ehs.uky.edu	Academic institution	Laboratory design / general infrastructure	Exploratory parameter framing only	1	1	http://ehs.uky.edu/docs/pdf/ohs_lab_ergonomics_0001.pdf
elss.cap.org	Professional / standards organization	Laboratory design / general infrastructure	Exploratory parameter framing only	1	1	https://elss.cap.org/elss/ShowProperty?nodePath=/UCMCON/Contribution%20Folders/DctmContent/education/OnlineCourseContent/2017/LAP-TLTM/checklists/cl-mol.pdf
learn.cap.org	Professional / standards organization	Laboratory design / general infrastructure	Exploratory parameter framing only	1	1	https://learn.cap.org/content/cap/pdfs/Competency_Model.pdf
microscopy.com	Other / mixed	Laboratory design / general infrastructure	Exploratory parameter framing only	1	1	http://www.microscopy.com/tu
mohap.gov.ae	Governmental body	Laboratory design / general infrastructure	Exploratory parameter framing only	1	1	http://www.mohap.gov.ae/Files/
msu.edu	Academic institution	Laboratory design / general infrastructure	Exploratory parameter framing only	1	1	http://www.msu.edu/uni
